# The Interaction Properties of the Human Rab GTPase Family – A Comparative Analysis Reveals Determinants of Molecular Binding Selectivity

**DOI:** 10.1371/journal.pone.0034870

**Published:** 2012-04-16

**Authors:** Matthias Stein, Manohar Pilli, Sabine Bernauer, Bianca H. Habermann, Marino Zerial, Rebecca C. Wade

**Affiliations:** 1 Molecular and Cellular Modeling Group, Heidelberg Institute for Theoretical Studies (HITS), Heidelberg, Germany; 2 Max Planck Institute for Dynamics of Complex Technical Systems, Magdeburg, Germany; 3 Max Planck Institute of Molecular Cell Biology and Genetics, Dresden, Germany; 4 Max Planck Institute for Biology of Ageing, Cologne, Germany; University of Cyprus, Cyprus

## Abstract

**Background:**

Rab GTPases constitute the largest subfamily of the Ras protein superfamily. Rab proteins regulate organelle biogenesis and transport, and display distinct binding preferences for effector and activator proteins, many of which have not been elucidated yet. The underlying molecular recognition motifs, binding partner preferences and selectivities are not well understood.

**Methodology/Principal Findings:**

Comparative analysis of the amino acid sequences and the three-dimensional electrostatic and hydrophobic molecular interaction fields of 62 human Rab proteins revealed a wide range of binding properties with large differences between some Rab proteins. This analysis assists the functional annotation of Rab proteins 12, 14, 26, 37 and 41 and provided an explanation for the shared function of Rab3 and 27. Rab7a and 7b have very different electrostatic potentials, indicating that they may bind to different effector proteins and thus, exert different functions. The subfamily V Rab GTPases which are associated with endosome differ subtly in the interaction properties of their switch regions, and this may explain exchange factor specificity and exchange kinetics.

**Conclusions/Significance:**

We have analysed conservation of sequence and of molecular interaction fields to cluster and annotate the human Rab proteins. The analysis of three dimensional molecular interaction fields provides detailed insight that is not available from a sequence-based approach alone. Based on our results, we predict novel functions for some Rab proteins and provide insights into their divergent functions and the determinants of their binding partner selectivity.

## Introduction

**Figure 1:**
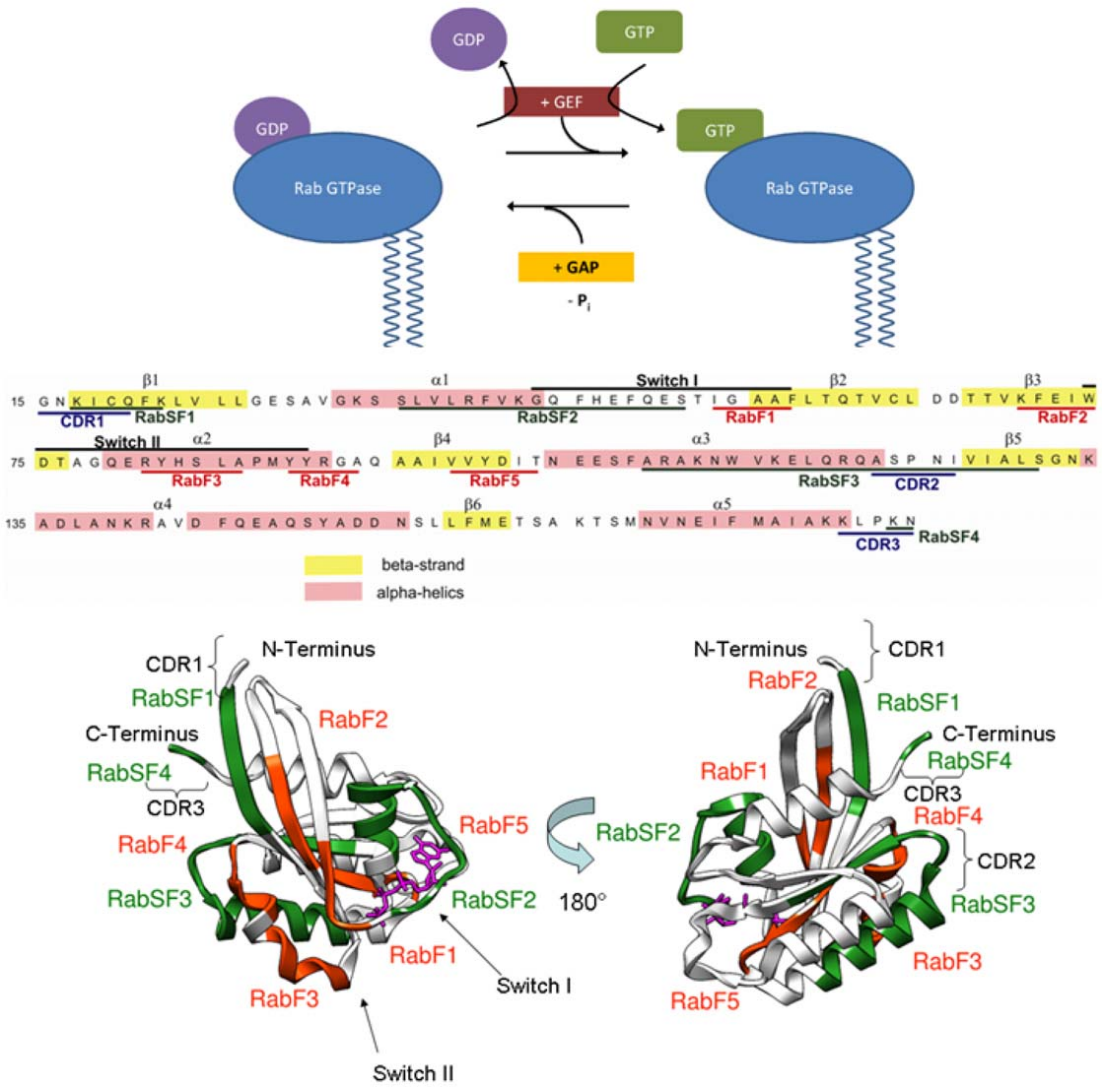
Rab GTPases as molecular switches. **A**: Schematic figure showing the interactions of a Rab-GTPase with its effector proteins. The Rab protein is geranyl-geranylated near the C-terminus to enable membrane binding. Guanine nucleotide exchange factor proteins (GEFs) accelerate the GDP to GTP exchange and thus convert the GTPase from its inactive into its active form. Guanine nucleotide activating proteins (GAPs) deactivate the Rab GTPase by facilitating the intrinsic GTP hydrolysis. **B**: Sequence and structural mapping of characteristic segments of Rab proteins. Rab family-specific (RabF1-RabF5) sequence segments that are distinct for Rab GTPases and distinguish them from other small GTPases of the Ras superfamily (Pereira-Leal and Seabra, 2001) are mapped onto the crystal structure of the Rab5A GTPase from *H. sapiens* in an active conformation (Terzyan et al., 2004) (displayed in orange-red). Rab subfamily (RabSF1-RabSF3) specific sequence segments are characteristic for subsets of the Rab GTPases in which each subfamily displays a high sequence identity (displayed in green) (Pereira-Leal and Seabra, 2000). The nucleotide is shown in pink. The Switch I and II regions, which undergo large nucleotide dependent conformational transitions, are labeled.

Rab proteins comprise the largest family of GTPases. Rab proteins localize to distinct intracellular membranes [1] where they act as regulators of organelle biogenesis, assembly and intracellular vesicle transport between different sub-cellular compartments (for reviews, see for example [2]–[4]). Like other members of the Ras superfamily, Rab proteins shuttle between the inactive (GDP-bound) and active (GTP-bound) forms (see **Figure 1A**).

Specific regulators enhance the rate of GDP to GTP exchange (guanine nucleotide exchange factors, GEFs) and GTP hydrolysis (guanine nucleotide activating proteins, GAPs).

Previous comparisons and classifications of Rab proteins were performed either at the amino acid sequence level using bioinformatics approaches [5]–[9] or at the functional level [2]. The cross-species mammalian sequence analysis of Pereira-Leal and Seabra yielded five Rab family (RabF) regions of the sequence that distinguish Rab proteins from other proteins of the Ras superfamily as well as four Rab subfamily specific (RabSF) regions that are highly conserved for each Rab subfamily [10]). In *Homo sapiens*, 11 subfamilies were defined based on a high degree of sequence conservation in the RabSF regions and co-segregation in phylogenetic trees [11]. The RabF and RabSF regions are mapped onto the Rab5 sequence and structure in **Figure 1B**. All Rab proteins share a common structural fold, which is composed of five α-helices and six β-strands connected by ten loops. Large conformational changes are associated with the switch I and II regions and depend on the nucleotide bound. Most of the RabF and RabSF regions fall in and around the switch regions (see Figure 1.) The RabF regions, along with the complementarity-determining regions (CDRs), are thought to provide the structural basis for the specificity and binding of regulatory proteins. The CDRs are regions that contact Rab-binding proteins in crystal structures [12] and are present near the N-terminus (CDR-1), in the middle of the sequence overlapping with RabSF3 (CDR-2), and near the C-terminus of helix-5 (CDR-3). Some of the Rab effector proteins have specific interactions with the C-terminal hypervariable domain (RabSF4, which is not considered in this study) whereas other effectors are independent of the hypervariable domain sequences and bind to multiple Rab proteins.

We here present and compare a sequence-based analysis of human Rab proteins with an analysis of their three-dimensional molecular interaction fields. Previous sequence-based analyses [6], [11], [13] of Rab proteins provided a phylogenetic tree of Rab proteins but this could not resolve ambiguities in the classification of some Rab proteins or some inconsistencies with experimental data. To improve the functional annotation of Rabs and obtain a better understanding of the determinants of their protein interactions, we carried out an extensive comparative analysis based on sequence, structure and molecular interaction fields. The analysis of molecular interaction fields (MIFs) by using the Protein Interaction Property Similarity Analysis (PIPSA) method [14], [15] yields results that complement those from sequence analysis due to the consideration of the three-dimensional protein fold and the fact that the interaction potential at a given point in space may be determined by the properties of amino acid residues that are not contiguous in sequence. PIPSA is therefore here applied to a set of human Rab proteins in order to cluster them according to their interaction and functional properties. Some related other approaches used a rotational invariant representation of the electrostatic potential [16] or additionally evaluate solvation free energy differences between alanine scan mutants and parent protein from calculated electrostatic potentials [17].

PIPSA has previously been applied to classify other protein families, for example, blue copper proteins [18], proteins containing WW domains [19], and proteins from the ubiquitination pathway [20], [21], according to their binding properties. The PIPSA approach has recently been extended to allow a quantitative comparison between protein electrostatic potentials and enzyme kinetic parameters for use in systems biology [22]–[24]. Here, PIPSA is applied to compare the electrostatic and hydrophobic interaction fields of human Rab proteins. For this application, a new feature has been introduced into PIPSA to permit the systematic scanning of MIFs in a spherical region around each surface residue to identify conserved and variable regions of the proteins.

In the next section, we describe the results of clustering 62 human Rab proteins according to sequence analysis. Then, the clustering of the Rab proteins resulting from PIPSA is described. We then discuss the implications of the clustering for the functional annotation of certain Rab proteins, for predicting their protein binding partners and for understanding mechanistic aspects of Rab function.

## Results and Discussion

### Protein sequence analysis

**Figure 2:**
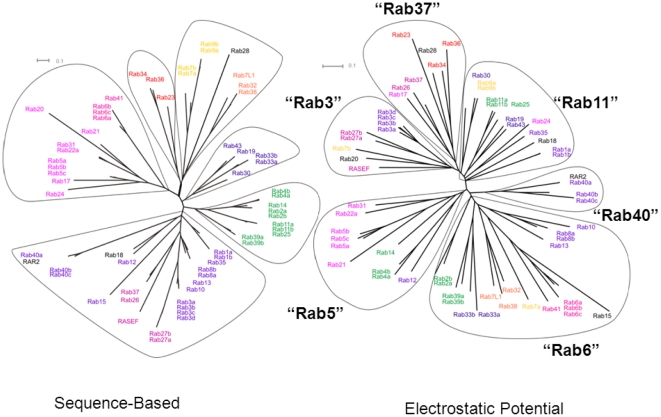
Clustering of human Rab proteins according to sequence and to electrostatic potential similarity. **A (left)**: Unrooted phylogenetic tree based on a Gblocked alignment of the sequences. The tree shows six subclusters. The color coding corresponds to the sequence-based phylogenetic analysis by Colicelli (Colicelli, 2004). For a phylogenetic tree derived from analysis of the full-length sequences, see File S1. **B (right)**: Epogram. The proteins are clustered according to their distance in electrostatic potential space, 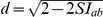, where SI_ab_ is the pairwise Hodgkin similarity index (Hodgkin and Richards, 1987) calculated for the complete protein skin. The electrostatic potential distance clustering suggests six subclusters with a different composition from the sequence-based analysis.

There are more than 60 different Rab proteins in humans [6]. We chose a set of 62 human Rab GTPases, omitting most proteins annotated as ‘putative’ and ‘Rab-like’ sequences. The phylogenetic tree computed from multiple-sequence alignment can be divided into six sub-clusters (‘leaves’), see **Figure 2A**. In general, the same assignment of Rab proteins to sub-clusters holds for the phylogenetic trees based on the Gblocked and the full-length alignment of sequences (see File **S1**).

The largest subcluster contains 23 members. The different forms of the Rab proteins 1a–b, 3a–d, 8a–b, 27a–b and 40a–c in this leaf are very close in sequence and this supports their annotation as isoforms. The average sequence identity of all 23 Rab proteins in this cluster is 56% with a standard deviation of ±13%. RASEF (also known as Rab45), a protein that contains an N-terminal calcium-binding EF-hand motif in addition to the GTPase domain, is a member of this sub-cluster.

The second largest sub-cluster, comprises 13 Rab proteins. It contains the Rab5a–c isoforms and two other Rab GTPases that have also been shown to associate with the early endosome, namely Rab21 and Rab22a. It also contains the Rab 6a–c isoforms and Rab41, which has 82% sequence identity with Rab6b, suggesting a functional annotation of Rab41 as a ‘Rab6-like’ Rab-GTPase.

The third largest subcluster contains 10 members, including the Rab 2, 4, 11, and 39 isoforms. The high sequence identity of 86% between Rab25 and Rab11a in the trimmed and 71% in the full sequence alignment suggests an annotation of the Rab25 sequence as a Rab11 family member. For Rab14, a sequence identity to Rab2a and 2b of 80% and to Rab4a and 4b of 77% in the trimmed alignment (65% and 66% in the full sequence alignment) make a clear functional assignment to either Rab2 or Rab4 difficult.

The fourth largest subcluster contains 8 members. It contains Rab7a and 7b, and Rab9a and 9b which are associated with the late endosome. The remaining two phylogenetic leaves, with 5 and 3 members, are not discussed here.

In a sequence analysis of the entire human Ras superfamily protein, Colicelli identified 4 large subfamilies of Rab GTPases [6]. The composition of our subcluster containing the Rab5 and Rab6 isoforms is identical to that of Colicelli, whereas there are some differences in the clustering of the other Rab sequences. For example, in our alignment, Rabs 39a, 39b, 25, 11a, 11b, 4a, 4b, 14, 2a, 2b form a leaf of their own whereas in Colicelli's study, they are part of a larger leaf.

Schwartz et al. [2] used Colicelli's superfamily alignment of the human Ras proteins to classify the Rab proteins into 14 subclusters and analyse their function and localization. All of the functionally annotated Rab proteins from Schwartz et al. are present in one of our six subclusters. One difference is the positioning of Rabs 27a and 27b in the same cluster as RASEF, Rab26 and Rab37 [2] whereas, in our alignment, the Rab27 proteins are very close to the Rab3a–d isoforms and at a larger distance to RASEF, Rab26 and Rab37, albeit in the same subcluster. The sequence identity between Rab3a and Rab27a is only 59% in the trimmed alignment (45% in the full sequence alignment) but the close functionality of Rab3 and Rab27 (for a review see [25]) is also supported by comparison of the electrostatic potentials (see below, Figure 2).

### Protein Electrostatic Potentials

To obtain the three-dimensional structural models necessary for PIPSA, we chose to model all the Rab-protein sequences analysed onto a single representative of the Rab GTPase protein family, namely Rab5, for which there are high resolution protein structures in both GDP- and GTP-bound forms available. We focused on the ‘core’ Rab domain and did not model the hypervariable domain since it is structurally not resolved. The use of one structural template for each PIPSA analysis ensured that the maximum possible values of the pairwise similarity indices were computed and differences identified were due to differences in sequence and were not affected by the noise that can arise from using several structural templates, e.g. for a flexible sidechain assigned to different rotameric states in different crystal structures although both rotamers could be occupied in the protein under physiological conditions.

We first compared the electrostatic potentials by computing pairwise similarity indices for the whole protein “skin”, a shell around the protein surface encompassing the most important region for interaction with potential binding partners. The results of the comparative analysis of the electrostatic potentials of the 62 human Rab GTPases are shown in **Figure 2B**. The relationship between the Rab proteins is displayed in an ‘epogram’ in which the proteins are clustered according to their distance apart in electrostatic potential space. The epogram has 6 subclusters, which we label by one representative member as the ‘Rab5’, ‘Rab6’, Rab40’, ‘Rab11’, ‘Rab37’ and ‘Rab3’ subclusters. In nearly all cases, members of Rab GTPase subfamilies (Rab GTPases with the same identifier number, e.g. Rab5a–c or Rab3a–d), cluster together. This shows that the Rab protein isoforms that are very close in primary sequence, exhibit very similar electrostatic potentials around their surfaces, and may thus be expected to bind to the same effector proteins and to perform a similar if not identical function.

The ‘Rab5 sub-cluster’ contains the early-endosome associated Rabs 5, 21, 22a, and 22b/Rab31. The same clustering occurs in the sequence analysis but the electrostatic potential-based subcluster additionally contains Rabs 4, 12 and 14. Rabs 4 and 14 also localize to early endosomes (see annotation in [2]). The conservation of electrostatic potentials in early endosome-associated Rab proteins may represent a molecular adaptation to the cellular and surface charges of early endosomes and can be used as a fingerprint to functionally annotate novel Rab proteins. The localization of Rab12 is controversial: it has been shown to bind to the Golgi apparatus [26], the perinuclear recycling compartment [27] and the late endosome [28]. The MIF analysis suggests that it should be tested whether Rab12 localizes to early endosomes.

At the sequence level, Rab14 is approximately equidistant from the Rab2a,b and the Rab4a,b proteins. When comparing the electrostatic potentials, Rab14 and Rab4a,b co-localize in the same Rab5-subcluster whereas Rab2a,b can be found in the adjacent Rab6-subcluster at a larger distance. This is in agreement with Rab14 and Rab4 GTPases both binding to early endosomes (see above) whereas Rab2 is functionally associated with the ER to Golgi transport (GO annotation).

The close relatedness of the interaction properties of Rabs 27a,b and 3a–d is matched by their electrostatic potential similarity as they cluster together in the Rab3-subcluster. Rab3 and Rab27 are both involved in the regulation of the final steps of the secretory pathway. Because of the structural relatedness of Rab27 and Rab3, it is sometimes not possible to discriminate between specific effector proteins and proteins that can mediate the action of both these GTPases [29].

**Figure 3:**
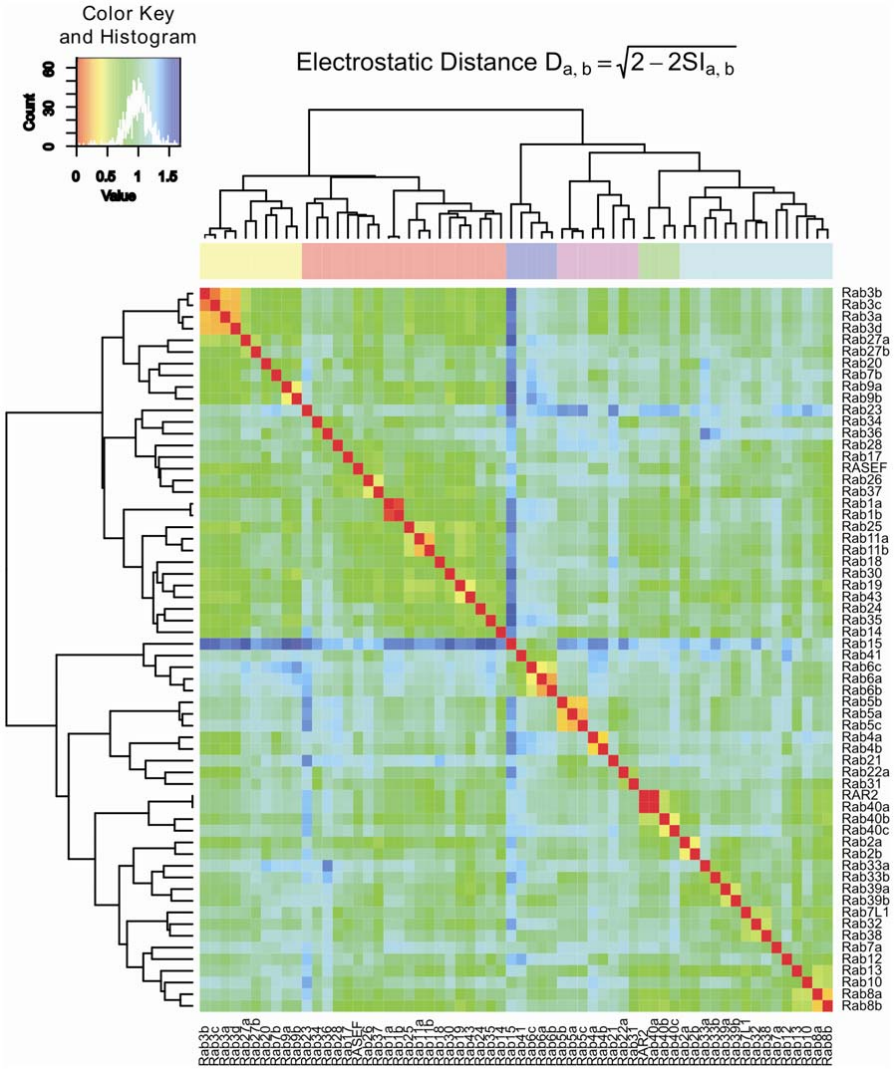
All pairwise comparison of electrostatic potentials in human Rab GTPases. Heat-map representation of the distance matrix of electrostatic potentials computed for the entire protein skins of human Rab proteins with the color code ranging from red (identical) to blue (dissimilar). The ordering of the Rab GTPases corresponds approximately to that of Figure 2B, right.

The coloured heatmap matrix for the all-pairwise comparison of electrostatic potentials shown in **Figure 3** provides another view of the relations between the Rab protein electrostatic potentials. Rab GTPase subfamilies, e.g. Rabs 5a–c or Rabs 3a–d, can be quickly identified as closely related due to their small distance in electrostatic potential (orange squares). Also, striking differences in the electrostatic potential of Rab15 and Rab23 compared to all the other human Rab GTPases are readily identified by their light- to dark-blue rows and columns.

The conservation of the electrostatic potential can be used as an indicator of the functional conservation of proteins. The high conservation of both amino acid sequence and electrostatic potential of Rab41 and Rab6, for example, gives strong support to the annotation of Rab41 as a Rab6-like protein. Although the former is not fully characterized, both localize to the Golgi apparatus and may have similar function [2]. On the other hand, Rab7a and 7b, which appear closely related at the sequence level, are separated by a large distance in electrostatic potential (Figure 3) and occupy positions in two different subclusters (Figure 2, right) and should not be referred to as “isoforms”.

### Rab7a and Rab7b have different interaction properties

**Figure 4:**
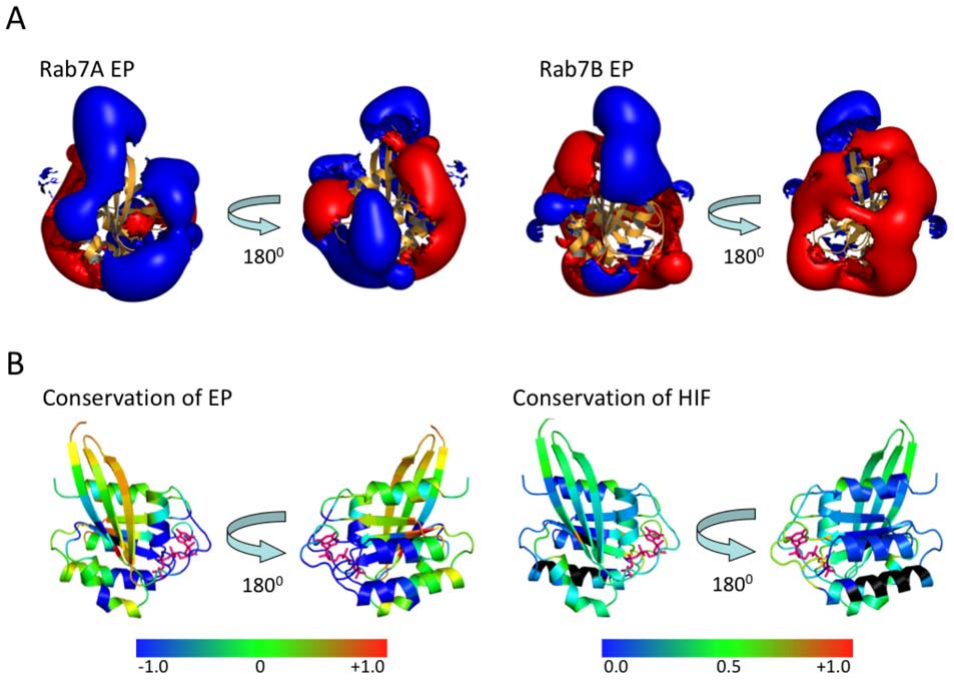
Differences in the electrostatic and hydrophobic interaction fields of Rab7a and 7b. **A**: Electrostatic isopotential contours at ±0.5 kT/e (blue: positive, red: negative) around cartoon representations of Rabs 7a and 7b. Each protein is shown in two views, one focusing on the switch I and II regions and the other rotated 180° about the vertical axis. **B**: Conservation of electrostatic and hydrophobic interaction fields mapped onto the protein crystal structure as a color gradient from blue (variable) through green to red (conserved) on a scale from −1 to +1 for electrostatic potentials and 0 to +1 for hydrophobic fields. The nucleotide is shown in pink. For the regions in black, no hydrophobic similarity index was computed due to high polarity.

One of the most striking findings from the PIPSA analysis is that Rab 7a and 7b are not part of the same subcluster but rather occupy positions far apart. Rab7a is a member of the ‘Rab6’ subcluster whereas Rab7b can be found in the ‘Rab3’ subcluster (Figure 2B). Figure 4A shows the electrostatic potentials of Rab 7a and 7b. There are large differences in these MIFs, in particular, around the functionally important nucleotide binding site and switch regions. The Hodgkin similarity index [30] (SI) between the two proteins is (only) 0.47. This is a low number for enzymes that are considered to be isoforms (see Methods for more details). Figure 4B shows the difference in electrostatic potentials and hydrophobic fields between the Rab7a and 7b proteins. The electrostatic potential of Rab7a is close to those of Rab41 and Rab6 (see Figure 2, right) whereas that of Rab7b is similar to Rab20. The striking differences in MIFs, as well as the moderate overall sequence identity of 51% in the full sequence alignment, suggest that Rabs 7a and 7b are functionally distinct, in contrast to their initial characterization [31]. The considerable differences in interaction properties of Rab7a and Rab7b are even more apparent when other structural templates are used (see File **S1**).

The variation of both electrostatic potentials and hydrophobic fields around functionally important regions indicate that Rab7a and Rab7b are not likely to share binding partners like GEFs, GAPs and effector proteins. The similarity of these Rab proteins at the sequence level alone is not a sufficient criterion for an annotation as isoforms and indeed they have been found to cluster separately in a phylogenetic analysis of Rab 7 and Rab 9 [32]. The conservation of MIFs, in particular around the switch regions, provides an additional criterion for the functional annotation of Rab proteins as ‘isoforms’ or ‘subfamilies’.

### Conservation of sequence and molecular interaction fields in human Rab GTPases

**Figure 5:**
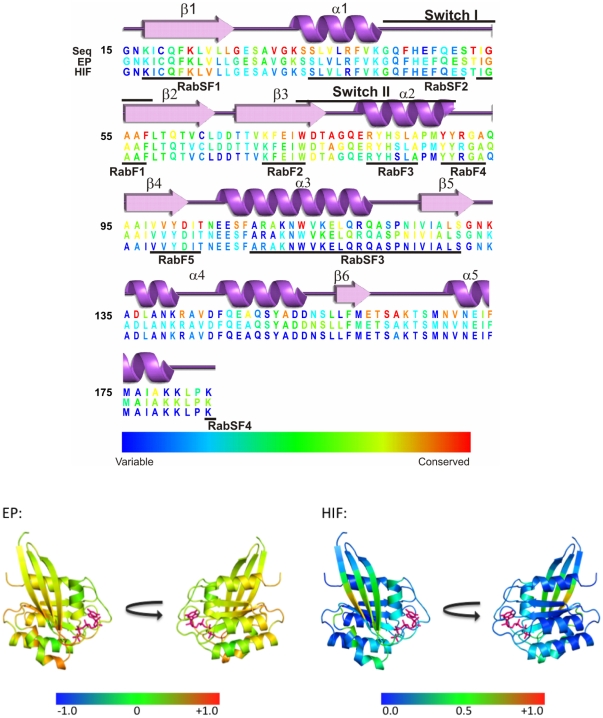
Conservation of sequence and molecular interaction fields in human Rab GTPases. **A**: Rab5a (PDB entry 1R2Q) secondary structure and amino acid residues colored from variable (blue), through intermediate (green) to conserved (red) according to conservation of sequence (Seq), electrostatic potential (EP) and hydrophic interaction field (HIF). **B:** Cartoon representation of Rab5a with bound nucleotide in stick representation (pink) and the similarity of the electrostatic (EP) and hydrophobic interaction fields (HIF) mapped onto the structures. The conservation scores of the corresponding residues are represented by a color gradient from blue (variable), through green to red (conserved). Each structure is shown in two views, one focusing on switch regions I and II and one rotated 180° about the vertical axis to highlight the helices and CDRs.

The sequence conservation of amino acid residues and the conservation of electrostatic potentials and hydrophobic fields in the vicinity of each residue were calculated for all human Rab proteins and are shown in **Figure 5**.

Figure 5A shows the conservation of amino acid sequences, electrostatic potentials and hydrophobic fields displayed as a color gradient from blue (variable) to red (conserved) of primary sequence letters. In terms of sequence, the most conserved residues mostly cluster around the switch II region (Trp74–Glu80). The most variable residues in human Rab GTPases are near the N- and C-termini.

The conservation of electrostatic potential and hydrophobic potential over all human Rab GTPases is mapped onto sequence in Figure 5A and onto a representative fold (PDBid: 1R2Q) in Figure 5B. For each residue, the pairwise SI values were averaged over all Rab protein pairs and then mapped onto the specific residue of the Rab5a sequence (shown in Figure 5 only for the active form). The conceptual thinking is different for comparing the similarity in terms of amino acid sequence and of 3D MIFs. Whereas substitution of a residue in a sequence has a local effect and can be clearly assigned to an individual residue, changes in the 3D MIFs *around* each residue originate from alterations of the properties, not only of the central residue, but also of the surrounding residues.

The highest conservation of the electrostatic potential is observed around the residues from the two switch regions (switch I and switch II). Gly54 of the switch I region and Ala77 of the switch II region are in close proximity to the γ-phosphate of the nucleotide and the coordinating Mg^2+^ ion. The electrostatic potential around Gly54 in the switch I region is the most conserved with an average SI of 0.81. It is followed by the electrostatic potential around Ala77 (SI 0.77) from the loop just preceding switch II. The most variable electrostatic potentials are found around residues which are part of the RabSF3 region (Phe108, Ala109, Arg110 and Ala111 (SI 0.1)).

The hydrophobic interaction field is a short range interaction. Thus, the hydrophobic fields in a radius of 15 Å around the Cα atoms are generally less conserved than the electrostatic potentials. The hydrophobic MIFs are most conserved around Lys 22 (SI 0.75), Leu23 (SI 0.78) and Val24 (SI 0.77) which are located close to the C-terminal end of the switch II region which constitutes RabF4 (residues 89–93) (see Figure 5A) and may stabilize the nucleotide-dependent conformation of the switch II region. The hydrophobic fields vary most around residues C-terminal to β4.

### Conservation of electrostatic and hydrophobic interaction fields in Rab subfamilies

**Figure 6:**
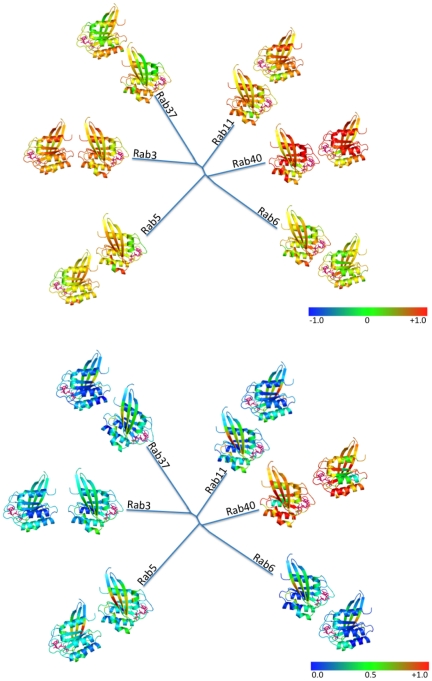
Structural mapping of molecular interaction field conservation in human Rab GTPase subclusters. Simplified Rab family tree in which representatives of subclusters from the electrostatic potential comparison (Fig. 2B) are displayed with the similarity of molecular interaction fields among the subcluster members. Cartoons are shown in two views, the ones close to the branch focus on the switch I and II regions and the ones further away are shown rotated by 180° around the vertical axis. The nucleotide is shown in a pink stick representation. The average of all pair-wise SI scores of all members of each subfamily is mapped onto the protein structure as a color gradient from blue (variable), through green (intermediate) to red (conserved) for **A**: electrostatic potential and **B**: hydrophobic interaction field. Positive HIF energies were neglected for similarity calculations and those regions are represented in black. For an alternative subclustering according to the sequence-based phylogentic tree, see also File S1.

From the epogram in Figure 2B, six subclusters (Rab3, Rab5, Rab6, Rab11, Rab37 and Rab40) were defined on the basis of distance in electrostatic potential space. The conservation and variability of electrostatic potentials and of hydrophobic fields in each of the six subclusters was investigated to elucidate subcluster-specific interaction properties. In **Figure 6**, the degree of variation and conservation of electrostatic potentials (Figure 6A) and hydrophobic fields (Figure 6B) within each subcluster in the active, GTP-bound form of Rab proteins is mapped onto the 3D protein fold.

The Rab40 subcluster is the smallest with just four members: the Rab40 isoforms and its close relative RAR2. It shows the highest degree of conservation of both electrostatic potentials (EP) and hydrophobic interaction fields (HIF) irrespective of the protein conformation (active or inactive).

The Rab6 subcluster is the largest, consisting of a diverse set of 19 Rab proteins. In the Rab6 subcluster, there is a high conservation of EP, in particular in the active form, around residues Ala77 and Gly78 of the switch II region, Gly32 of the phosphate-binding loop and Gly54 of RabF1 (part of the switch I region). In the inactive form, the similarity index around Ala77 and Gly78 (0.89 vs. 0.86) is almost unchanged but around Gly54 drops from 0.87 in the active form to 0.74 in the inactive form. In the inactive form, Val99 (in the RabF5 region) becomes the residue with the most conserved electrostatic potential.

The Rab5, Rab3 and Rab11 subclusters also show a large degree of conservation of electrostatic potentials, independent of the conformational states of the switch regions.

The Rab37 subcluster, though smaller in size, shows less conservation of EP than the previously mentioned subclusters. Large variations in electrostatic potential are observed for β-strands 2 and 3 but the electrostatic potential is conserved in α-helix 3 in both the active and inactive conformations.

For Rab3, Rab11 and Rab40 subclusters, the electrostatic potentials in switch region II and α-helices 4 and 5 are more conserved in the active (GTP-bound) conformation of the Rab protein than in the GDP-bound form.

For hydrophobic interaction fields, the conservation is significantly lower than for the electrostatic potentials. The hydrophobic interaction fields are only well conserved in the Rab40 subcluster (in both the active and inactive forms). This difference can be explained by the fact that HIFs are short-range and particularly sensitive to local changes in conformation. Conservation in the Rab40 subcluster is due to the high level of sequence identity (greater than 70% for the full protein sequences).

If the clustering is done on the basis of similarity of sequence (phylogenetic tree in Figure 2A) rather than electrostatic potential (File **S1**), the level of conservation of EP and HIF is lower. The greatest conservation of EP and HIF can be found in the Rab43 and Rab2 subclusters, which have a relatively small number of members.

### Nucleotide dependent conformational changes have large effects on interaction properties

**Figure 7:**
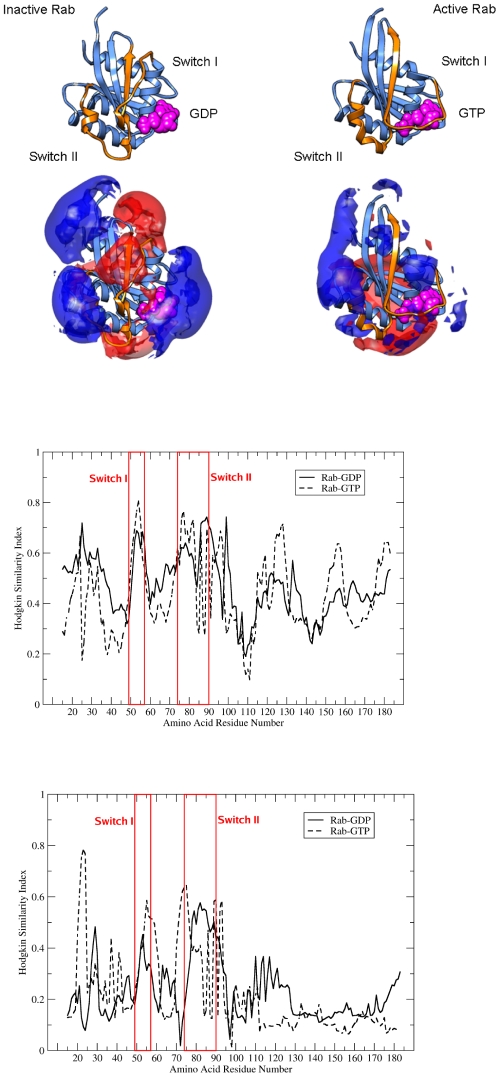
Nucleotide-induced conformational changes in molecular interaction fields. **A:** Differences in conformation and electrostatic potential between the inactive GDP-bound and active GTP-bound forms of human Rab5a. Isocontour plots at levels of +1 kT/e (blue) and −1 kT/e (red). The largest conformational change is associated with the switch II region (formation of a C-terminal alpha-helical region) and the switch I region (tight binding of GTP). **B:** The Hodgkin similarity index for the electrostatic potential **(top)** and the hydrophobic interaction field **(bottom)** within a radius of 15 Å around the Cα atoms of the inactive (GDP-bound) and active (GTP-bound) human Rab proteins is plotted against residue number.

Long-range electrostatic and short-range hydrophobic interactions are important determinants of the specificity and affinity of GEF, GAP and effector binding to Rab proteins. The switch function of Rab proteins between inactive (GDP-bound) and active (GTP-bound) states is accompanied by large conformational changes, in particular of the switch I and II regions. The systematic comparison of MIFs of human Rab family proteins reveals differences in regional conservation between the active and inactive forms (see **Figure 7**).

The nucleotide-dependent conformational change of Rab proteins is accompanied by a change in the electrostatic potential, in particular around the residues of the switch I and the interswitch regions (see Figure 7A). Figure 7B (top) shows the conservation of the electrostatic potential in the inactive and active forms of human Rab proteins. The mean SI values are similar in the two states: 0.47 for GDP- and 0.44 for GTP-bound states. However, local nucleotide-dependent changes can be observed. In particular for the N-terminal residues 15–47, the Hodgkin SI values in the GTP-bound form are lower. A large degree of conservation of electrostatic potential in the GTP-bound form can be detected for the switch I region and the starting residues of the switch II region which might act as a conformation-specific flag for subsequent effector binding.

Figure 7B (bottom) shows the conservation of hydrophobic interaction fields in inactive and active human Rab proteins. The mean hydrophobic MIF is nearly identical for the active and inactive forms of Rab (0.22 and 0.23). In particular, hydrophobic interaction fields around the C-terminal region of β 1 (residues 20–25) and the spatially close regions of β-strands 2 and 3 (residues 54–75) are more conserved in the active GTP-bound form than in the inactive form. The higher conservation of the hydrophobic interaction fields in of the N-terminal half of the Rab sequences (residues 15–72) in the GTP-bound form suggests an involvement of hydrophobic interactions in stabilizing the active form of Rab proteins.

The complementarity-determining regions (CDRs) are responsible for a tight interaction between Rab proteins and their binding partners. In the active form, the CDRs form a pocket with a non-conserved hydrophobic interaction field. Adjacent to this pocket, there is a well conserved hydrophobic region and the residues that contribute most are Leu23 (SI 0.78), Val24 (0.77) and Lys22 (0.75) of strand-1 (yellow patch in Figure 5B right). An invariant hydrophobic triad of residues has been proposed to stabilize the conformation of the switch regions [33]; these residues are in a region showing high conservation of hydrophobic interaction field in the active conformation only. A significant change in HIF between active and inactive forms for residues of the hydrophobic triad, i.e., Trp74 (from 0.64 to 0.16), Tyr89 (from 0.59 to 0.5) and Phe57 (from 0.51 to 0.33) can be observed. Apparently, the nucleotide-induced change in amino acid side chain orientations leads to changes in hydrophobic fields of this triad of residues which thus convey information about the bound nucleotide and act as a recognition motif for active Rab effectors [34], [35]. Merithew et al. [33] have shown that it is this structural plasticity of the hydrophobic triad that is responsible for effector specificity of Rabphilin3A towards Rab3A but not Rab5C. We were able to show that it is not a simple side chain re-orientation of residues of the hydrophobic triad that accounts for effector specificity, but rather a more complex, non-local change in hydrophobic interaction fields (there is an intrinsic increase in conservation of hydrophobic fields in all Rab proteins in the active, GTP-bound state).

### The switch I and II regions in subfamily V Rab GTPases

Across all small GTP-binding proteins, the guanine nucleotide binding site, the Mg^2+^ binding sites and the phosphate-binding loop (P-loop) are conserved and largely retain the same conformation whether GDP or GTP is bound. The switch I and switch II regions are not conserved in sequence and undergo large structural changes. The residues of the switch regions coordinate the γ-phosphate group of GTP but not GDP. Upon GEF binding, the switch I and switch II regions undergo large conformational changes again. In particular, the switch II region interacts strongly with the GEF.

The recently published crystal structure of the complex of the nucleotide-free subfamily V Rab21 and the GEF Rabex-5 provides structural insights into the Rab-GEF interaction [36]. In particular, interactions between Ser55 (corresponding to Ala57 in Rab5A; switch I region), and Arg80 (corresponding to Arg82 in Rab5A, switch II region) accounted for the hydrogen bonding interactions between the Rab-GTPase and the GEF.

We focused specifically on the comparison of electrostatic potentials of subfamily V Rab GTPases for this region (see Figure 2 and File **S1**). We investigated whether we could rationalize specific GEF-Rab recognition by comparing the electrostatic potentials in spheres of 15 Å radius around the Cα atoms of Ala57 and Phe58 in the switch I and Arg82 and Tyr83 in the switch II regions.

In spheres around Ala57 and around Phe58, the similarity (SI) of Rab5a–c and Rab21 is 0.76 and 0.70, respectively, while for Rab22a the corresponding values are only 0.27 and 0.30. The electrostatic potentials of the Rab5 isozymes around Ala57 and Phe58 are conserved. Mutational studies revealed that these residues are critical for a rationalization of the different Rabex-5 nucleotide exchange activities for subfamily V Rab-GTPases Delprato et al. [37]. The catalytic efficiency of Rabex-5_132–391_ for Rab5 and Rab21 was nearly indistinguishable, whereas that of Rab22a was two orders of magnitude lower. Clearly, the electrostatic potential around these switch I residues is an important factor for understanding the orders of magnitude difference in Rabex-5 GEF activities of subfamily V Rab GTPases [36], [37]. For spheres of radius 15 Å around Arg82 and around Tyr83 of the switch II region, the electrostatic potential is more conserved among the subfamily V Rab GTPases (details are given in the File **S1**).

Rab proteins that are characteristic for early endosomes (Rab5, Rab21, Rab22) show highly conserved electrostatic potentials around switch II and subtle differences around switch I and the interswitch regions. Rab proteins that are associated with late endosomes (like Rab7) show strikingly different electrostatic potentials and thus probably do not share any exchange factors with early endosome Rab GTPases. This difference in electrostatic potentials may also be an adaptation to the varying membrane composition between early and late endosomes.

### GEF recognition of switch II followed by switch I

Vetter and Wittinghofer have discussed the mechanism of GEF action as a push-and-pull mechanism in which switch I is pushed out of its conformation upon GEF binding and switch II is pulled towards the nucleotide binding site [38]. From our analysis, we suggest that first the nucleotide-bound state is recognized by the conformation of the switch II region (with conserved electrostatic potential) by the exchange factor and pushed towards the nucleotide. Then, GEF specificity and differences in exchange kinetics become important when the GEF flips over and binds to the switch I-interface region which is then pulled out of its GDP-bound conformation and releases GDP.

**Figure 8:**
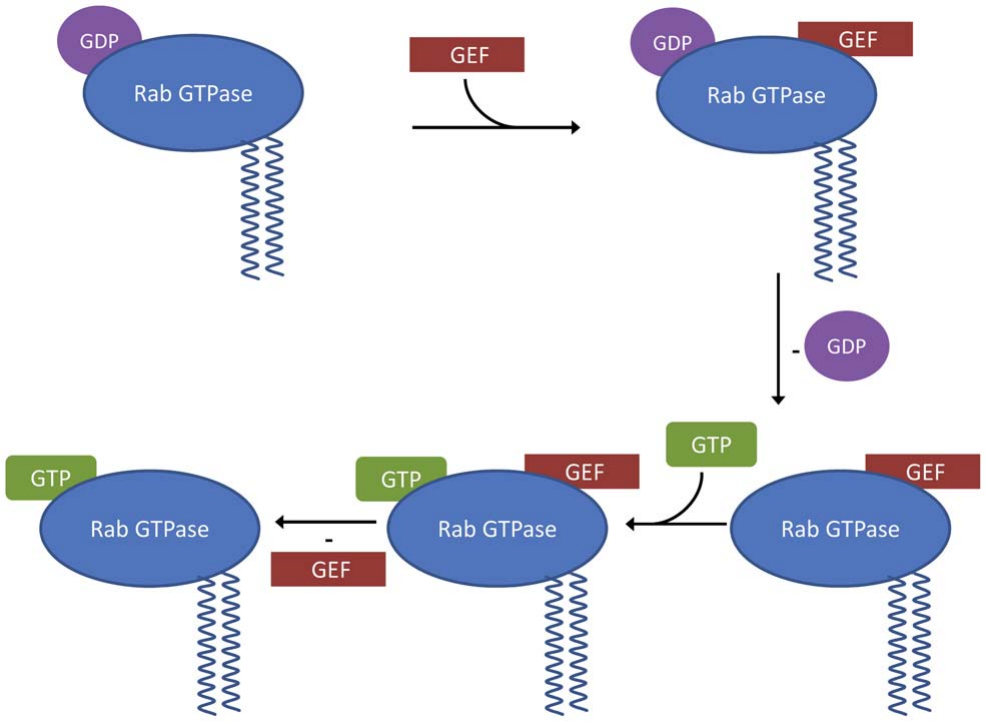
Proposed sequential action of the guanine nucleotide exchange factor (GEF) on geranyl-geranylated Rab-GTPases. The GEF destabilizes the ternary GDP-Rab-GEF complex and GDP is released. The nucleotide-free Rab and the GEF form a stable binary complex. Subsequent GTP binding to the nucleotide-free Rab again destabilizes the ternary complex, the GEF is released and the Rab is left in its active GTP-bound state.

The sequence of events regarding nucleotide exchange in Rab proteins has been the subject of recent discussion. Guo et al. presented evidence for an allosteric, sequential mechanism of nucleotide exchange [39]. In this mechanism, the GTPase can form stable binary complexes with either nucleotides or with exchange factors but less stable ternary complexes with both GEF and the nucleotides simultaneously bound. In the ternary complex, GDP and GEF are bound orders of magnitude less strongly than in the binary complex. This reduction of affinity leads to a release of GDP or GEF at elevated rate constants. Since, at the cellular level, GTP is in excess of GDP, the nucleotide free GTPase will predominantly bind to GTP, which then leads to a dissociation of the GEF (see Figure 8).

The family of human Rab GTPase proteins performs a wide range of different physiological functions. The large expansion of the Rab family in multicellular organisms reflects the higher complexity of cellular processes and a more specialized, tissue- and cell-specific role [11]. The large number of different Rab proteins, however, complicates their formal functional assignment. Here, we have analysed conservation of sequence and of molecular interaction fields to cluster and annotate the human Rab proteins.

The clustering of the Rab proteins according to their molecular interaction fields gives a picture of Rab proteins that is not available from a sequence-based analysis. For example, Rabs 3, 10, 13 and 8 appear very similar at the sequence level but the electrostatic potentials of the Rab 3 isoforms are significantly different from those of the others. We can therefore predict that it is unlikely that Rab 3 isoforms share any effector proteins with Rabs 8, 10 and 13. Also, the discrimination between Rab proteins that were previously annotated as Rab 7a and 7b is pronounced at the level of molecular interaction fields, both electrostatic and hydrophobic. This detailed analysis of human Rab protein properties delivers insight into the conservation and variability of molecular interaction fields within the largest family of Ras-type proteins. From our results, we can not only assign novel functions to some of the proteins, but also provide insights into their divergent functional behavior. The results can be used to guide the screening of Rab protein effector binding *in vitro* as well as other experiments to investigate the function of Rab proteins.

## Materials and Methods

### Sequence database searches

Sequence similarity searches were carried out using PSI-BLAST [40] from the stand-alone NCBI BLAST application using standard settings and filtering turned on. The sequence of Rab1 (NP_004152) was used to find all members of the Rab-family of GTPases in the human genome (protein sequence database, February 2008 release). Most Rab-like proteins, as well as the Rab42 (partial sequence) and Rab44 (ambiguous variations in sequence annotation) proteins were omitted from the analysis. For functional annotation, we used the review by Stenmark [41] if not otherwise stated and Gene Ontology. [42]

### Generation of multiple sequence alignments

ClustalW [43] and PROBCONS [44] were used to generate multiple sequence alignments. The resulting alignments were sent to Gblocks [45] to remove badly aligned regions.

### Phylogenetic analysis

Maximum likelihood (ML) trees were inferred from the multiple sequence alignments using PhyML [46] with the following parameters (if not default): 1000 bootstrap steps using PhyML generated pseudodatasets from the original datasets; transition ratio and proportion of invariable sites = estimated; number of substitution rate categories = 8; gamma distribution parameter = estimated. Distance-based trees were generated using *protdist* and *fitch* from the PHYLIP [47] package, using default parameters and inferring bootstrap values with *seqboot*-generated data sets (1000 steps). Branch lengths of the consensus tree were calculated by resubmission of the distance metric and the consensus tree to the fitch package (http://evolution.genetics.washington.edu/phylip/doc/consense.html).

### Protein Structural Modeling

Modeller8v2 was used with a modified refinement protocol to generate models of the protein structures based on one template protein structure [22], [48], [49]. For details of the model generation procedure, see [22]. The PDB entry 1R2Q (Rab5a from *H. sapiens* in complex with a GTP-analogue at 1.05 Å resolution) was used as the template to model the GTP-bound state of Rab proteins [50]. The amino acid sequence identity to this template was between 100% for Rab5a and 29% for Rab32 with an average sequence identity of 42%. For the active form, the PDB entry 1TU4 (human Rab5a with GDP) was used as a template. The degree of amino acid conservation is the same. All 62 human Rab sequences were modelled onto Rab5a protein structures. The crystal structure templates cover a range of residues from Gly15 to Lys183 and thus represent the intrinsic ‘core’ Rab structure. Particular Rab-specific N- and C-terminal extensions, for example, the mostly unstructured C-terminal hypervariable region (of 35 to 40 amino acid residues in length), which is partially responsible for the association of Rab proteins with specific target membranes [51], are thus not considered in this investigation. Comparative modeling to a single protein template structure was used in order to maximize the degree of similarity in three-dimensional molecular interaction fields (see below) and to exclude influences from different crystallization conditions. The reported numerical values are thus upper limits of possible similarity between human Rab proteins. The accuracy of this procedure has been shown before [22], [52]

### Calculation of electrostatic potentials

Electrostatics are a key determinant of macromolecular function (for some reviews, see [53], [54]. Protein electrostatic potentials were calculated by solving the finite difference linearized Poisson-Boltzmann equation using UHBD [55]. A protein dielectric constant of 4.0 and an exterior dielectric constant of 78.0 with an ionic strength of 50 mM were used. The grid dimensions were 110×110×110 Å^3^ with a spacing of 1 Å. The dielectric boundary of the proteins was defined by the molecular surface accessible to a spherical probe of 1.4 Å radius, representing a water molecule.

### Calculation of hydrophobic interaction fields

Protein hydrophobic interaction fields were calculated using the GRID program, version 22b [56]. Hydrophobic interaction energies at each point on a grid with a grid spacing of 0.5 Å were calculated as the sum of Lennard-Jones energy (ELJ) and water entropy (WENT) minus hydrogen bond energy (EHB) terms using the ‘DRY’ probe. For grid points in highly polar regions, hydrophobic energies are positive and automatically reset to zero, so hydrophobic interaction field similarities cannot be calculated for these regions.

### PIPSA

PIPSA [14], [22], [57] was used to quantitatively compare the protein MIFs within a skin of 3 Å thickness and defined with a probe of radius 2 Å. Comparisons were performed for the complete skins and also locally for spheres of 15 Å radius around the Cα atoms of each residue. The Hodgkin similarity index (SI) [30] 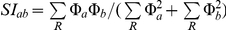 and distance d = 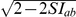 were used to compare the MIFs. The similarity index SI_ab_ ranges from −1 (anticorrelated) to +1 (fully correlated) interaction fields and the distance d from 2 (anticorrelated; large distance) to 0 (fully correlated; small distance).

### Systematic scanning of regional conservation of interaction fields in human Rab proteins

Pair-wise similarity indices were calculated in a sphere of 15 Å radius around the Cα atom of each residue. Conservation scores were calculated by averaging the SI values of all Rab protein pairs considered for each residue. The scores in spheres for which the dry probe delivered only positive interaction energies were set to zero.

## Supporting Information

File S1 **Supporting information figures and table.**
(DOC)
