# Supplementary material for: The Interaction Properties of the Human Rab GTPase Family – A Comparative Analysis Reveals Determinants of Molecular Binding Selectivity
Source: PLoS One. 2012 Apr 16;7(4):e34870. doi: 10.1371/journal.pone.0034870 (PMC3327705; doi:10.1371/journal.pone.0034870)
Supplement: File S1 — Supporting information figures and table. (DOC) [file pone.0034870.s001.doc]

**Supporting Information**

**The Interaction Properties of the Human Rab GTPase Family – A Comparative Analysis Reveals Determinants of Molecular Binding Selectivity**

### Matthias Stein^1,2§^, Manohar Pilli^1^, Sabine Bernauer^3^, Bianca H. Habermann^3,4^, Marino Zerial^3^, Rebecca C. Wade^1§^

^1^Molecular and Cellular Modeling Group, Heidelberg Institute for Theoretical Studies (HITS), Schloss-Wolfsbrunnenweg 35, 69118 Heidelberg, Germany.

^2^Max-Planck-Institute for Dynamics of Complex Technical Systems, Sandtorstrasse 1, 39104 Magdeburg, Germany.

^2^Max Planck Institute of Molecular Cell Biology and Genetics, Pfotenhauerstrasse 108, 01307 Dresden, Germany.

### ^3^Max Planck Institute for Biology of Ageing, Gleueler Straße 50a, 50931 Cologne, Germany.


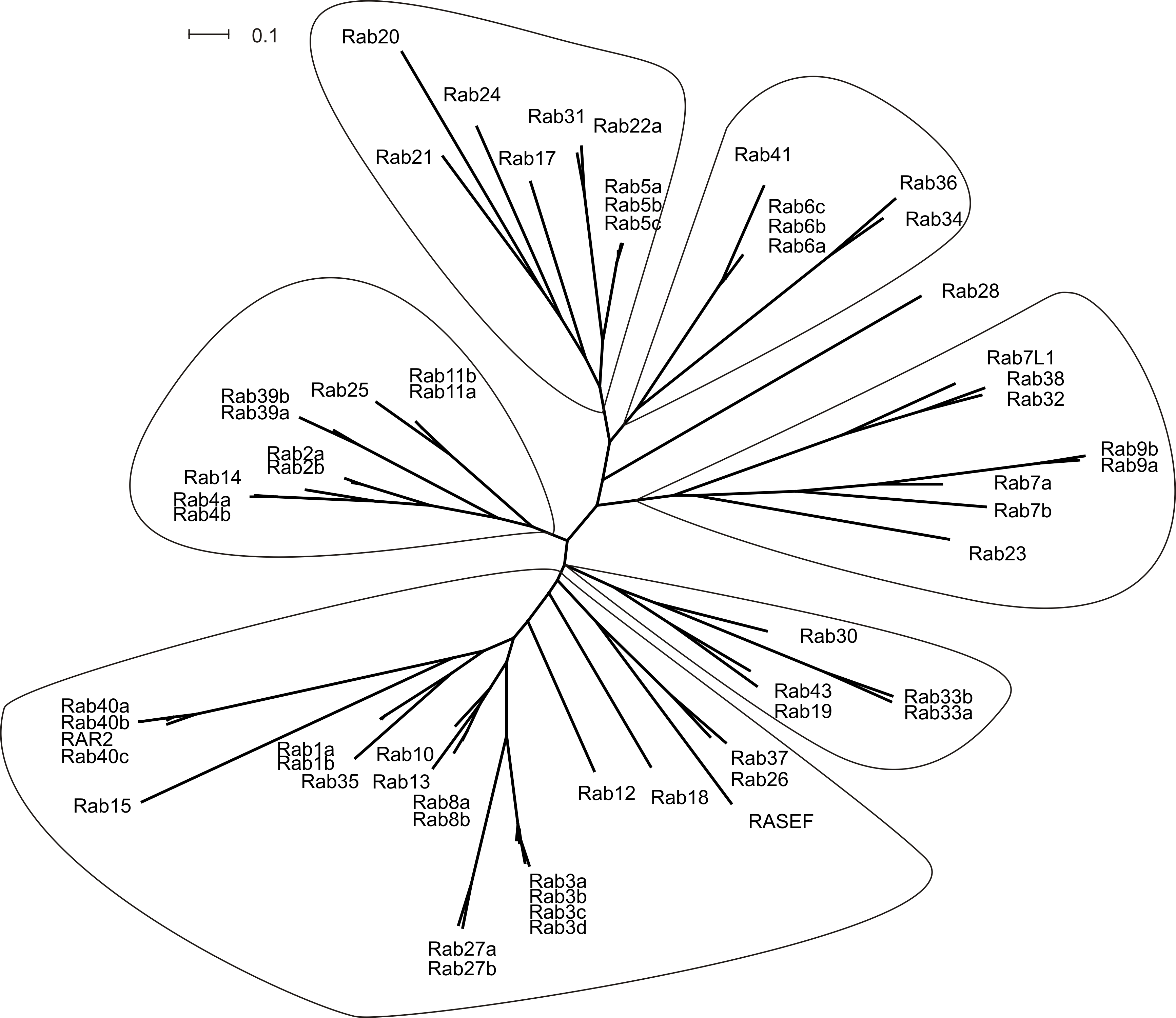


**Figure S1**

Full-length sequence tree of human Rab GTPases. The ClustalW alignment was subjected to 1000 bootstrap iterations with PhyML


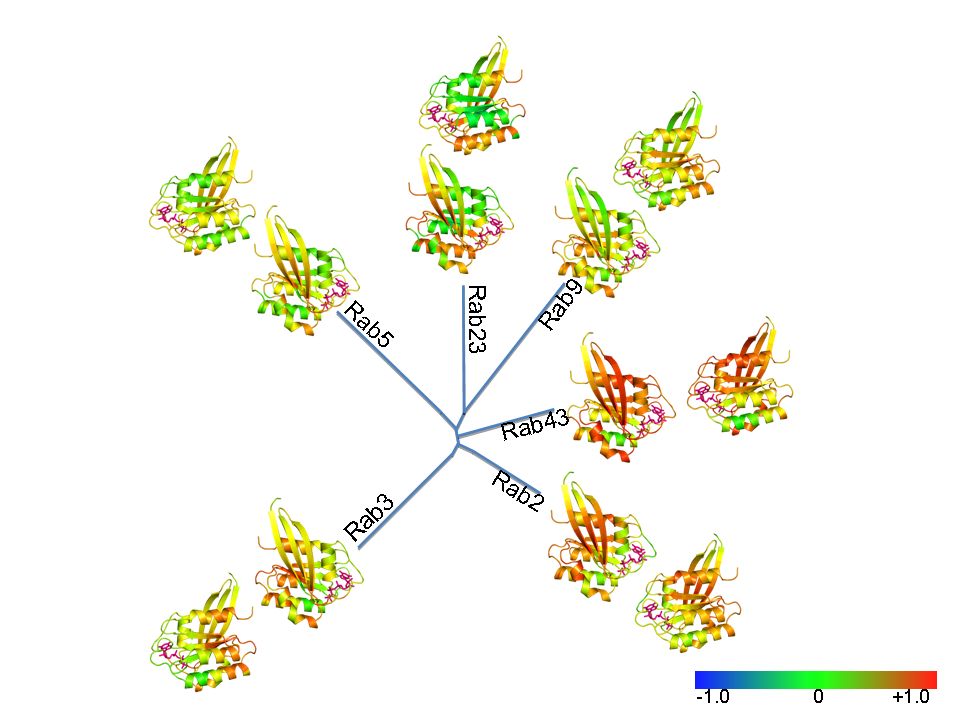


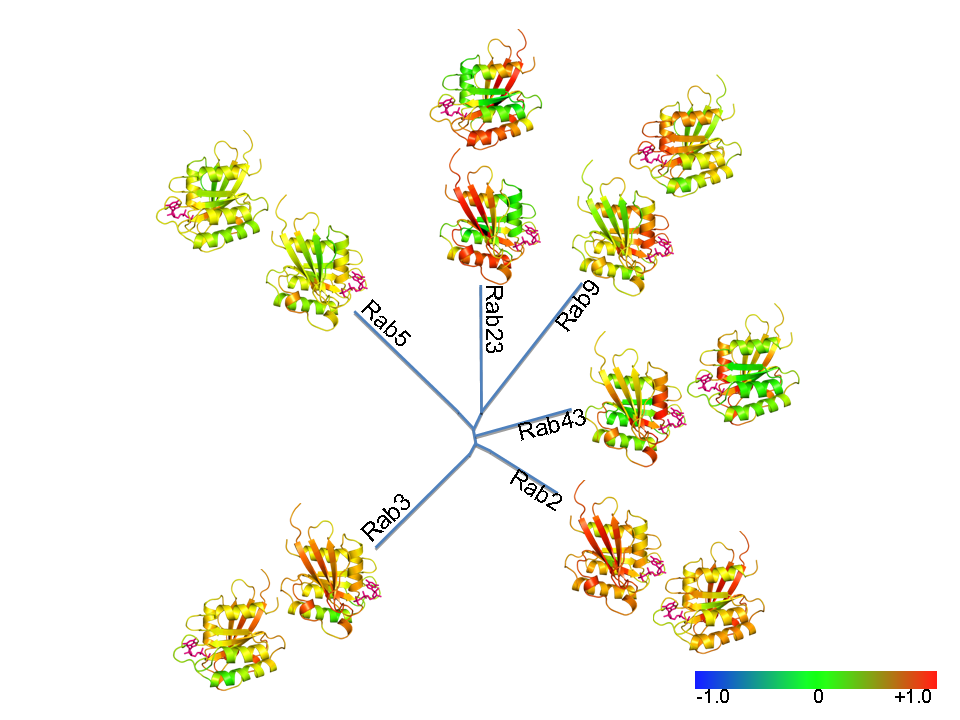


**Figure S2**

Conservation of electrostatic potentials in alternative Rab subfamily clustering according to sequence-based phylogenetic tree. The Rab GTPase is shown in a cartoon representation. The cartoons are shown in two views. The average of the pair-wise SI scores of all members of each subcluster for each of the 169 residues (number corresponds to the residues in all the modeled structures) are mapped to the cartoon as a color gradient from blue (-1), through green to red (+1).

**A (top)**: Active conformation. The Rab5a template (1R2Q) is shown as a cartoon and bound GTP analogue as a pink stick model.

**B(bottom)**: Inactive conformation. The Rab5a template (1TU4 chain A) is shown as a cartoon and the bound GDP as a pink stick model.

**
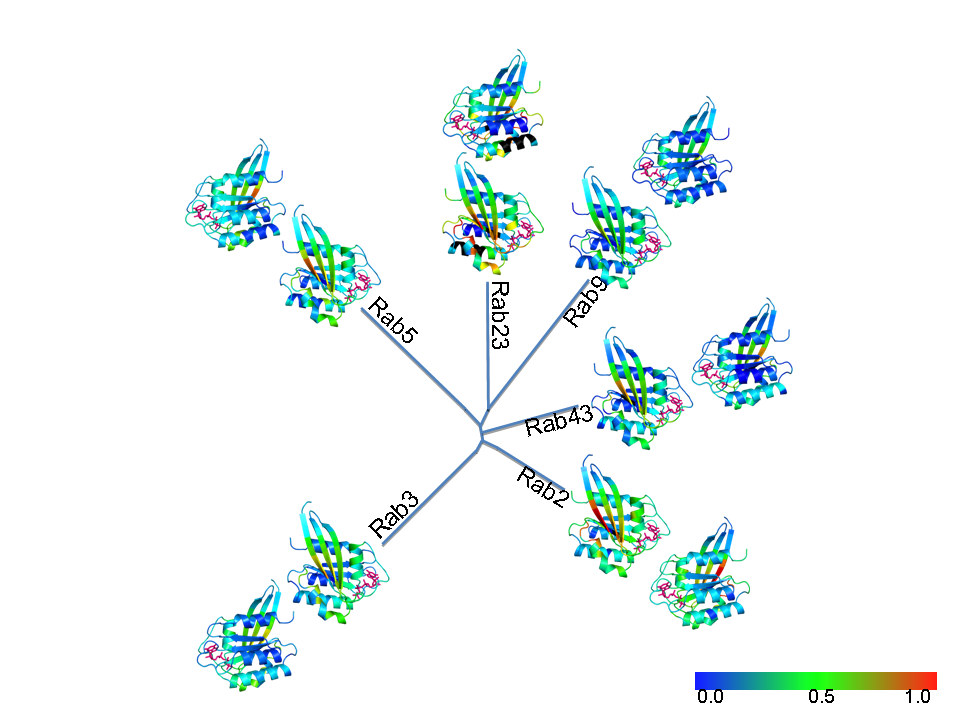
**

**
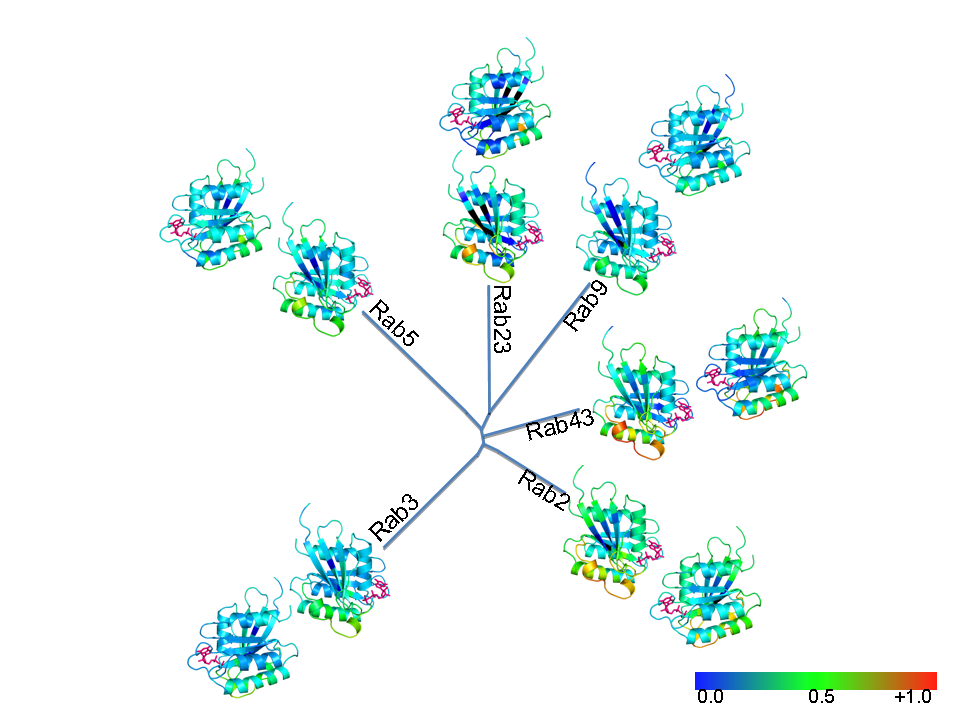
**

**Figure S3**

Conservation of hydrophobic interaction fields in alternative Rab subfamily clustering according to sequence-based Rab phylogenetic tree. The Rab GTPase is shown in a cartoon representation. The cartoons are shown in two views. The average of pair-wise SI scores of all members of each subcluster for each of 169 residues (number corresponds to the residues in all the modeled structures) are mapped to the cartoon as a color gradient of from blue (0), through green to red (+1).

**A (top)**: Active conformation. The Rab5a template (1R2Q) is shown as a cartoon and bound GTP analogue as a pink stick model.

**B (bottom)**: Inactive conformation. The Rab5a template (1TU4 chain A) is shown as a cartoon and bound GDP as a pink stick model.

**Table S4**

Hodgkin similarity indices and differences in electrostatic potentials for selected residues of a subset of human Rab GTPases. Comparison of the degree of conservation of amino acid sequences and similarities of electrostatic potentials for a selected subset of human Rab GTPases relative to GTP-bound Rab5a.

| **Rab GTPase** |  | **Hodgkin Similarity Index of Electrostatic Potentials**  **(Differences in Electrostatic Potentials in kcal mol^-1^ e^-1^)*** | | | | | |
| --- | --- | --- | --- | --- | --- | --- | --- |
|  | **Amino Acid Sequence Identity** | **Full Protein** | **Switch I Ala57** | **Switch I**  **Phe58** | **Switch II Arg82** | **Switch II**  **Tyr83** | **CDR 2 Asn126** |
| **Rab5a** | 100 % | 1.0 | 1.0  (0.0) | 1.0  (0.0) | 1.0  (0.0) | 1.0  (0.0) | 1.0  (0.0) |
| **Rab5b** | 88 % | 0.96 | 1.0  (-0.02) | 1.0  (-0.02) | 1.0  (-0.01) | 1.0  (-0.01) | 0.99  (-0.02) |
| **Rab5c** | 95 % | 0.97 | 1.0  (-0.03) | 1.0  (-0.04) | 0.98  (-0.07) | 0.98  (-0.07) | 0.98  (+0.02) |
| **Rab21** | 44 % | 0.68 | 0.76  (+0.03) | 0.70  (+0.06) | 0.84  (-0.14) | 0.92  (-0.07) | 0.52  (+0.21) |
| **Rab22a** | 51 % | 0.67 | 0.27  (+0.34) | 0.30  (+0.36) | 0.86  (-0.01) | 0.87  (+0.12) | 0.93  (-0.004) |
| **Rab7a** | 41 % | 0.18 | 0.71  (+0.01) | 0.75  (+0.08) | 0.48  (-0.17) | 0.44  (+0.21) | 0.47  (+0.48) |
| **Rab7b** | 34 % | 0.49 | 0.71  (+0.02) | 0.66  (+0.12) | 0.76  (+0.15) | 0.76  (+0.21) | 0.44  (+0.49) |

*Φ(Rab5a) – Φ(Rab_i_)
